# Transmission Efficacy and Plasticity in Glutamatergic Synapses Formed by Excitatory Interneurons of the Substantia Gelatinosa in the Rat Spinal Cord

**DOI:** 10.1371/journal.pone.0008047

**Published:** 2009-11-30

**Authors:** Sónia F. A. Santos, Liliana L. Luz, Peter Szucs, Deolinda Lima, Victor A. Derkach, Boris V. Safronov

**Affiliations:** 1 Instituto de Biologia Molecular e Celular (IBMC), Universidade do Porto, Porto, Portugal; 2 Laboratório de Biologia Celular e Molecular, Faculdade de Medicina, Universidade do Porto, Porto, Portugal; 3 Vollum Institute, Oregon Health and Science University, Portland, Oregon, United States of America; Tokyo Medical and Dental University, Japan

## Abstract

**Background:**

Substantia gelatinosa (SG, lamina II) is a spinal cord region where most unmyelinated primary afferents terminate and the central nociceptive processing begins. The glutamatergic excitatory interneurons (EINs) form the majority of the SG neuron population, but little is known about the mechanisms of signal processing in their synapses.

**Methodology:**

To describe the functional organization and properties of excitatory synapses formed by SG EINs, we did non-invasive recordings from 183 pairs of monosynaptically connected neurons. An intact presynaptic SG EIN was specifically stimulated through the cell-attached pipette while the evoked EPSCs/EPSPs were recorded through perforated-patch from a postsynaptic neuron (laminae I-III).

**Principal Findings:**

We found that the axon of an SG EIN forms multiple functional synapses on the dendrites of a postsynaptic neuron. In many cases, EPSPs evoked by stimulating an SG EIN were sufficient to elicit spikes in a postsynaptic neuron. EPSCs were carried through both Ca^2+^-permeable (CP) and Ca^2+^-impermeable (CI) AMPA receptors (AMPARs) and showed diverse forms of functional plasticity. The synaptic efficacy could be enhanced through both activation of silent synapses and strengthening of already active synapses. We have also found that a high input resistance (R_IN_, >0.5 GΩ) of the postsynaptic neuron is necessary for resolving distal dendritic EPSCs/EPSPs and correct estimation of their efficacy.

**Conclusions/Significance:**

We conclude that the multiple synapses formed by an SG EIN on a postsynaptic neuron increase synaptic excitation and provide basis for diverse forms of plasticity. This functional organization can be important for sensory, i.e. nociceptive, processing in the spinal cord.

## Introduction

The spinal SG is an important part of the nociceptive processing system. It is mostly formed by local excitatory and inhibitory interneurons, some of which relay the primary afferent inputs to projection neurons in lamina I [1]–[6]. The SG is frequently considered in terms of the gate control theory [7] emphasising the role of inhibitory circuits, and perhaps for this reason, organization of excitatory circuits was less studied [6]. There are however a number of reports indicating importance of SG EINs. Independent studies using *in situ* hybridization, immunocytochemistry and paired electrophysiological recordings showed that the majority of SG neurons are excitatory, while inhibitory neurons represent a minority [4], [8], [9]. Immunocytochemistry and EM studies have also revealed that excitatory synapses in the superficial dorsal horn express both GluR2-containing CI-AMPARs and GluR2-lacking CP-AMPARs [10]–[13]. The contribution of CP-AMPARs to transmission may increase under chronic pain conditions [14]–[17]. The superficial dorsal horn also expresses Ca^2+^-calmodulin dependent protein kinase II [18], [19] involved in plasticity induction upon Ca^2+^ entry in glutamatergic synapses [11], [20], [21]. Therefore, non-NMDAR synapses of SG EINs [4] might undergo plasticity based on CP-AMPARs. The physiological evidences for plasticity in the SG EIN synapses and for involvement of CP-AMPARs in transmission from SG EINs have not been reported so far.

Here we combined paired recording, computer simulation and biocytin-labelling to study functional organization and activity-dependent modification of glutamatergic synapses of SG EINs. Special care was taken to choose experimental conditions for recording from pairs of monosynaptically connected cells. It is known that the efficacy of synaptic transmission depends on passive membrane properties of a postsynaptic neuron, i.e. R_IN_ and membrane time constant, which vary with the composition of intracellular recording solution. R_IN_ in SG neurons is above 1 GΩ when measured in the whole-cell mode with pipette solutions containing strong Ca^2+^ chelators [4], [22], [23] or in the perforated-patch mode [4] which prevents dialysis of cytoplasmic factors and Ca^2+^ [24], [25]. R_IN_ is lower when measured in whole-cell with Ca^2+^-chelator-free solutions (158 MΩ, [26]). On the other hand, the presence of a strong Ca^2+^ chelator in the presynaptic neuron may affect synaptic release [27]. For these reasons, we did paired recordings under conditions preserving the cytoplasmic composition in both neurons. Recording from the postsynaptic neuron was done in the perforated-patch mode while the intact presynaptic SG EIN was specifically stimulated through a cell-attached pipette. The cell-attached pipette was also used for the SG EIN labelling with biocytin [28] for the analysis of its axon terminals.

We show that axon of an SG EIN forms multiple functional synapses on dendrites of a postsynaptic neuron. In many cases, EPSPs evoked by stimulating an SG EIN initiated postsynaptic spikes. The EPSCs were carried through both CP- and CI-AMPARs, and showed different forms of plasticity. The synaptic efficacy could be increased through the activation of silent synapses and through the increase in the strength of active synapses. We also show that a high R_IN_ (>0.5 GΩ) in a postsynaptic neuron is necessary for resolving the distal dendritic inputs. It is concluded that the multiple synapses of an SG EIN on a postsynaptic neuron increase efficacy of transmission and provide basis for diverse forms of plasticity.

## Methods

### Preparation

Rats (2-6-weeks) were sacrificed in accordance with the national guidelines (Direcção Geral de Veterinária, Ministério da Agricultura) after the anaesthesia by intraperitoneal injection of Na^+^-pentobarbital (30 mg/kg) and subsequent check for lack of pedal withdrawal reflexes. The procedure has been approved by the institutional ethics committee (Comissão de Ética do Instituto de Biologia Molecular e Celular). Transverse slices (200–300 µm) were prepared from the lumbar spinal cord as described [22], [23]. Neurons were visualized in slices using the oblique LED illumination technique [28], [29]. The SG was identified in the dorsal horn as a light band of about 60 µm thickness in its intermediate region as previously described [4]. The marginal 20 µm layer separating the SG from the white matter was considered as lamina I.

### Solutions and Junction Potentials

ACSF contained (in mM): NaCl 115, KCl 3, CaCl_2_ 2, MgCl_2_ 1, NaH_2_PO_4_ 1, NaHCO_3_ 25 and glucose 11 (pH 7.4; bubbled with 95% O_2_/5% CO_2_). The perforated-patch pipettes were filled with solution containing (in mM): NaCl 5, K-gluconate 145, HEPES 10 (pH 7.3 adjusted with KOH, final [K^+^] was 148 mM) and amphotericin B (100 µg/ml). Amphotericin B forms pores permeable to small monovalent ions Na^+^, K^+^ and Cl^−^, but not to Ca^2+^ and large cytoplasmic anions [25], [30]. In pipette solution, we used impermeable gluconate as the major anion and set [Cl^−^] to its cytoplasmic level (5 mM) to have negligible Donnan equilibrium junction potential [24], [30]. The liquid junction potential [31], [32] between the pipette and bath solutions was 15.5 mV; it was corrected for in all recordings. The stimulating pipettes were filled with ACSF. D-AP5, GYKI-52466, SYM 2081 and IEM 1460 were from Tocris. All the remaining chemicals were from Sigma.

### Biocytin Labelling

In the labelling experiments, a postsynaptic neuron was filled by biocytin in the whole-cell mode using pipette containing (in mM): KCl 3, K-gluconate 150.5, MgCl_2_ 1, BAPTA 1 and HEPES 10 (pH 7.3 adjusted with KOH, final [K^+^] was 160.5 mM) and 1% biocytin. An intact presynaptic SG EIN was filled (without rupturing the patch membrane) through the cell-attached stimulating pipette [28] which contained 500 mM NaCl and 1.5% biocytin. Increased osmomolarity improved biocytin efflux from the cell-attached pipette and filling the neuron. Labelled neurons were processed and reconstructed as described [28]. Due to different experimental conditions, these recordings were not included in the statistics based on perforated-patch recordings.

### Computer Simulation

Simulations were done using NEURON software [33], [34] and the SG neuron model from [23] (http://senselab.med.yale.edu/ModelDb, 62285). This basic model had R_IN_ of 1.6 GΩ, the membrane time constant of 91 ms and the dendrite electrotonic length of 0.68 λ [23]. Voltage-dependent Na^+^ and K^+^ conductances were set to zero, while the electrotonic EPSC/EPSP propagation was analysed. A two-state kinetic scheme was used to model the synaptic response. The current flowing through the channels in the synapse activated by transmitter release (I_S_) was described by the following equation:
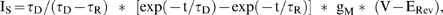
where τ_R_ is the conductance rise time constant, τ_D_ is the conductance decay time constant, g_M_ is the maximum conductance, V is the membrane potential and E_Rev_ is the reversal potential (set to 0 mV). According to our Results, the constants τ_R_ and τ_D_ were set to 0.5 ms and 5 ms, respectively. R_IN_ was reduced from 1.6 GΩ to 1 GΩ, 500 MΩ, 300 MΩ, 200 MΩ and 100 MΩ by increasing the specific passive membrane conductance from 1.1*10^−5^ S/cm^2^ to 1.9*10^−5^ S/cm^2^, 4.8*10^−5^ S/cm^2^, 1.05*10^−4^ S/cm^2^, 2.1*10^−4^ S/cm^2^ and 6.6*10^−4^ S/cm^2^, respectively. Simulations were done for the resting/holding potential of −70 mV. The synapse location and g_M_ are specified in figures.

### Electrophysiology

Recording pipettes were pulled from thick-walled glass (BioMedical Instruments, Germany) and fire-polished (resistance, 3–5 MΩ). The stimulating pipettes were also fire-polished (resistance, 12–24 MΩ). The amplifier was EPC-10-Double (HEKA, Lambrecht, Germany); its voltage-follower circuitry was used in current-clamp (CC). The low-pass filter frequency in voltage-clamp (VC) was 3 kHz. The frequency of digitization was 10 kHz. Offset potentials were compensated before seal formation. The series resistance was 6–19 MΩ. R_IN_ was measured in CC using hyperpolarizing currents (10 pA, 500 ms). Resting potential was measured with the balanced amplifier input [35]. The perforated-patch recordings were done 20–30 min after establishing the gigaseal to allow membrane perforation and reduction of the access resistance. Recordings were done at 22–24°C.

Membrane noise was measured in the perforated-patch mode using 100 ms traces (digitalized at 0.1 ms; repeated 10 times for each potential) resembling those used for the recording of synaptic responses. Noise was analyzed in the frequency range between 10 Hz (determined by the trace length) and 3 kHz (low-pass filter frequency in VC). The noise was presented as the root mean square (r.m.s.) noise, or σ, which is also identical to the square root of the variance [36]. The intrinsic noise of the amplifier measured before the experiment was <90 fA.

Monosynaptic connections of SG EINs were identified by paired recording as described in [4]. The present study, however, demanded long-lasting recordings with continuous cell-attached stimulation of the presynaptic neuron. As we have found, the pulse (100 nA; 1 ms) used in [4] was too strong for continuous stimulations and provoked damage of the presynaptic neuron before the end of the protocol. Therefore, we first tested how the pulse amplitude could be reduced to provide reliable and non-damaging stimulation for longer periods of time.

**Figure 1:**
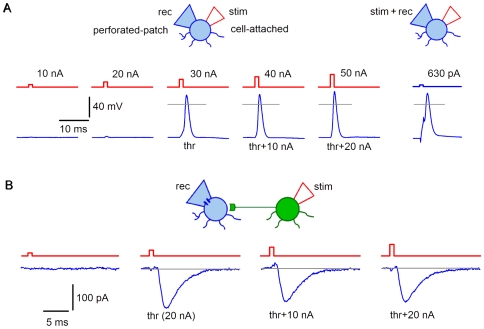
Adjustment of the stimulation strength. A, an SG neuron current-clamped via the perforated-patch recording pipette was stimulated by the current injection (1 ms; 10 nA increment) through the cell-attached pipette. The first spike was evoked at 30 nA (threshold). Right, spike evoked in the same neuron by a 1 ms current injection through the recording pipette. It is seen that the initiation phase of the spike evoked by the cell-attached stimulation does not show voltage distortions which typically appear when one electrode is used for both stimulation and recording. Grey line, 0 mV. B, the strength of the cell-attached stimulation for the SG EIN in a connection was adjusted by recording the evoked EPSCs (−80 mV). For this connection, the threshold was at 20 nA, and therefore, the stimulation at 40 nA (threshold+20 nA) was used throughout the experiment.

An SG neuron was kept in CC at −70 mV through the perforated-patch recording pipette (Fig. 1A). The tip of the stimulating pipette was attached to the same neuron with application of a slight suction. The neuron was stimulated through the cell-attached pipette by currents of increasing amplitude (10 nA, increment). The first stimulation eliciting spike was considered as threshold. The threshold stimulation increased by 20 nA (threshold +20 nA) reliably evoked spikes in all SG neurons tested (n = 10). In paired recording (Fig. 1B), a connection was first identified using a 100 nA stimulation [4], but then the pulse amplitude was appropriately adjusted. The postsynaptic neuron was in VC at −80 mV while the presynaptic SG EIN was stimulated at 10 nA increment. The first pulse eliciting EPSC was considered as threshold. The ‘threshold +20 nA’ pulse was used in the following experiment (n = 183). For different connections, it ranged between 30 nA and 100 nA (in most cases, 40–50 nA) and provided reliable stimulation of the presynaptic neuron for periods up to 1 hour.

**Figure 2:**
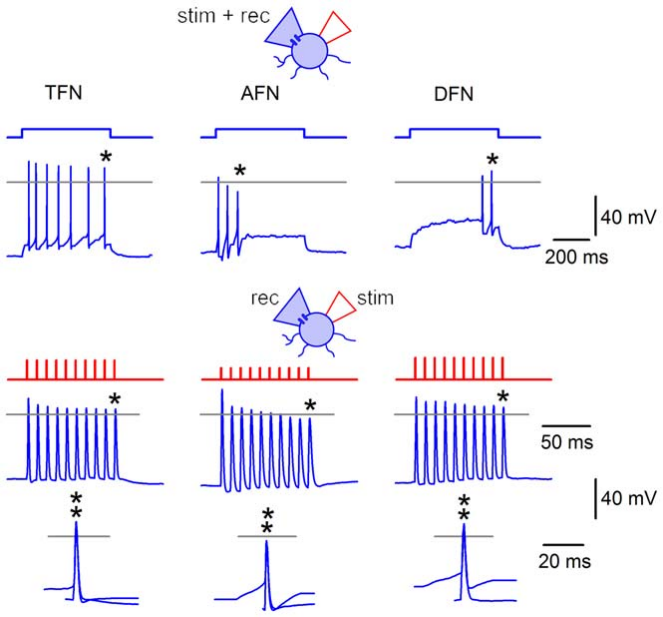
Stimulation of SG neurons at 100 Hz. The perforated-patch pipette and the cell-attached pipette were placed on the soma of an SG neuron. Depolarizing currents (500 ms; 40 pA) were first injected through the perforated-patch and the neuron was classified according to its firing pattern as tonic-firing neuron (TFN), adapting-firing neuron (AFN) or delayed-firing neuron (DFN) [4], [35]. Following classification, the same neuron was stimulated through the cell-attached pipette at 100 Hz (10 pulses). Each pulse (1 ms) had the ‘threshold+20 nA’ amplitude. The last spikes in trains (asterisks) elicited by these stimulation techniques are shown superimposed on the bottom.

It was also tested whether the cell-attached stimulation can evoke a high-frequency firing needed for plasticity-induction protocols. Three types of SG neurons with distinct firing patterns and thresholds were described in our experiments [4], [35]. In 15 neurons (n = 5, for each type), we recorded spikes induced by a train of 10 pulses (1 ms) applied at 100 Hz (Fig. 2). In all neurons tested, the cell-attached stimulation evoked firing at 100 Hz. Thus, the ‘threshold+20 nA’ pulses could be used for repetitive stimulation of all types of SG neurons.

All numbers are given as mean ± SEM unless otherwise mentioned. The parameters were compared by paired Student's *t* test.

## Results

We identified 221 monosynaptic connections with presynaptic SG neurons by testing ∼900 presumably presynaptic neurons. The probability of finding connections in this study (∼25%) was higher than in [4] (∼10%) because of the implementation of the oblique LED illumination technique [28], [29] allowing selection of neurons from a larger pool of visible cells. Inhibitory connections (n = 38) were not subjected to analysis. The excitatory connections (n = 183) form the basis of the present study. Of 183 postsynaptic neurons, 16 were in lamina I, 144 in the SG and 23 in lamina III. The resting potential, R_IN_ and the slowest membrane time constant in postsynaptic neurons were −74.3±0.5 mV (n = 183), 1.77±0.08 GΩ (n = 183) and 79.6±3.5 ms (n = 162), respectively.

The experiments were done to study 1) the threshold of spike initiation in an SG neuron, 2) the pharmacological properties of synapses formed by SG EINs, 3) the kinetics of EPSCs and their change with the synapse location along the dendritic tree of the postsynaptic neuron, 4) how the experimental conditions lowering R_IN_ in the postsynaptic neuron affect both the resolution and the efficacy of distal synaptic inputs, 5) the efficacy of EPSPs to evoke spikes in a postsynaptic neuron, 6) whether synapses of SG EINs can show short- and long-term plasticity, 7) whether an SG EIN forms single or multiple synapses on a postsynaptic neuron, and 8) the release probability in the individual synapse.

### Spike Initiation in an SG Neuron

**Figure 3:**
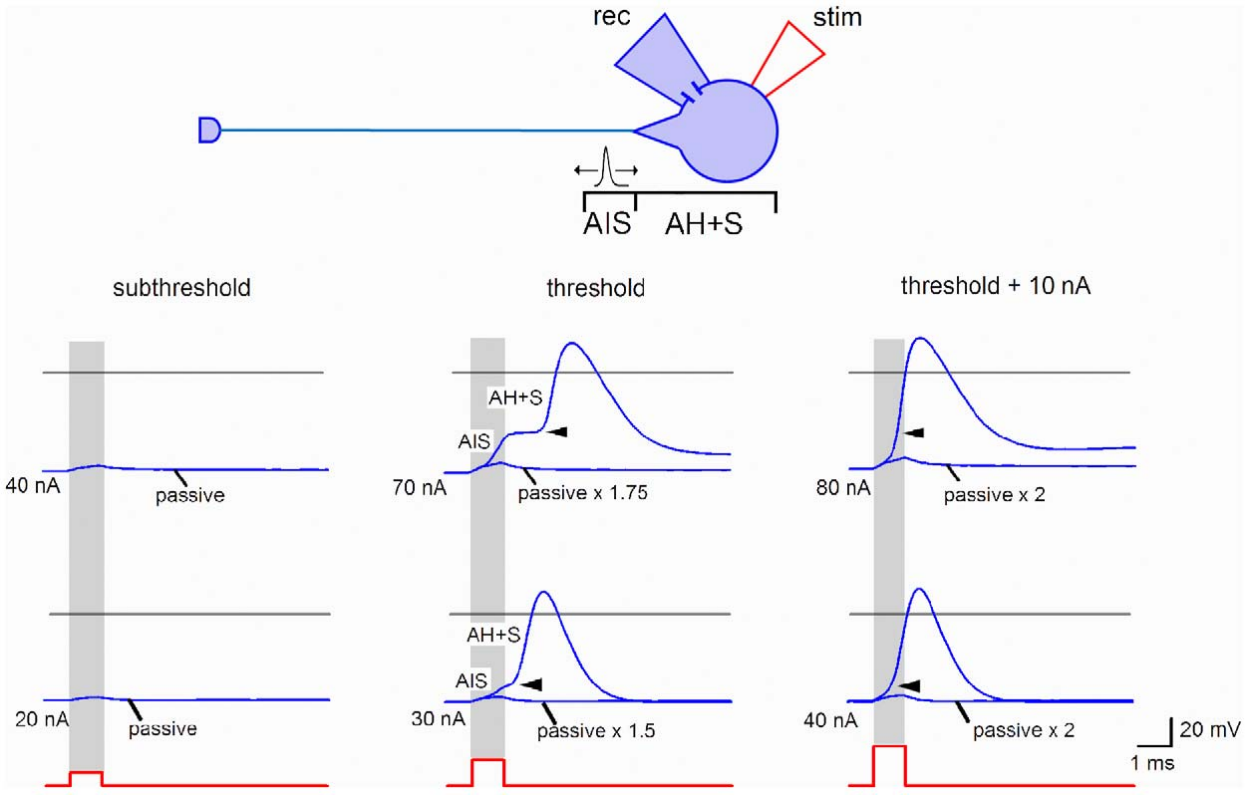
Spike initiation in an SG neuron. Stimulating (cell-attached) and recording (perforated-patch) pipettes were placed on the soma of an SG neuron. Stimulation (1 ms) increased with a 10 nA increment. Subthreshold, the membrane responses were considered as passive. Threshold, the AIS and AH+S components could be best distinguished (transitions are indicated by arrowheads). The AIS component was initiated within the 1 ms of the stimulation. The passive response was multiplied by the corresponding scaling factor. Threshold+10 nA, the interval between the AIS and AH+S components became shorter. The AIS component was completely generated within the 1 ms of the stimulation. Two SG neurons shown have different proportions between the amplitudes of the AIS and AH+S components and different kinetics of transitions. Horizontal grey lines, 0 mV. Resting potentials were about −70 mV.

The estimation of firing threshold in a neuron is needed for evaluation of efficacy of synaptic transmission. In addition, correct estimation of the spike initiation time in the axon initial segment (AIS) of the presynaptic neuron is critical for determining the latency of the postsynaptic response. These estimations can hardly be done in experiments where one electrode is used for both stimulation and recording (Fig. 1A, right), since injections of large currents needed to evoke spike by short pulses (1 ms) cause capacitance transients and voltage drop on the access resistance which, even after compensation, distort recording at the moment of spike initiation. Undistorted spike could however be recorded when stimulation was delivered via the cell-attached pipette (Fig. 1A). Therefore, we studied the spike initiation in an SG neuron stimulated via the cell-attached pipette (Fig. 3). At subthreshold level, a small passive intracellular response was observed which increased linearly with stimulation intensity. This response was appropriately scaled and superimposed on the suprathreshold traces, to estimate the contribution of the passive component. At the firing threshold, the amplitude of the passive component was 10.7±1.7 mV (n = 10) when measured from the resting potential. This passive depolarization was sufficient to trigger a spike consisting of two components separated by an inflexion point (arrowhead, Fig. 3). These components were similar to those described in the classical studies of motoneurons [37]–[39], which attributed the fast and slow components to the spike invasion to the AIS and the soma, respectively. Since the soma of dorsal horn neurons has low density of Na^+^ channels and is not able itself to generate spikes [40], [41], the two components of the spike in SG neurons could be explained by the spike generation in the AIS and its antidromic invasion to the tightly coupled electrotonic domain consisting of the axon hillock and the soma (AH+S) [42], [43]. At a threshold (Fig. 3), the AIS component of the spike was initiated during the 1 ms stimulation, and the interval between the initial phases of the AIS and AH+S components was 0.99±0.18 ms (n = 9; range 0.4–1.9 ms; in 1 of 10 cells the components could not be distinguished). The amplitude of the AIS component was 15.1±2.1 mV (n = 9; measured from the passive component) and varied from cell to cell as shown in Fig. 3 (threshold). At ‘threshold +10 nA’ stimulations, the AIS component was completely activated during the stimulation pulse.

Thus, short depolarizations (∼10 mV) induced by the cell-attached stimulation were sufficient to elicit spike in the AIS. From the AIS, the spike propagated in two directions: orthodromically, towards the axon terminals, and antidromically, to the soma. For this reason, we considered the end of the stimulation pulse, where the AIS spike has already been generated, as a moment from which the EPSC latency has to be measured. These experiments have also indicated that the traditional measurement of the latency from the peak of the somatic spike can overestimate the true latency by adding the time needed for the antidromic spike propagation from the AIS to the soma (up to ∼2 ms). This overestimation can vary from spike to spike, since the time interval between the AIS and AH+S components varies with stimulation intensity and alterations in the membrane potential [42], [44].

### Pharmacological Properties of Synapses Formed by SG EINs

Pharmacological properties of EPSCs were studied in 68 connected cell pairs. The postsynaptic receptor antagonists were applied for 30–60 s (perfusion rate, 1 volume of the experimental chamber per 5 s), but if a complete block was not achieved by the end of the application, its duration was increased, in some cases to 20–21 min (to allow drug diffusion to deeper synapses in the spinal cord slices).

**Figure 4:**
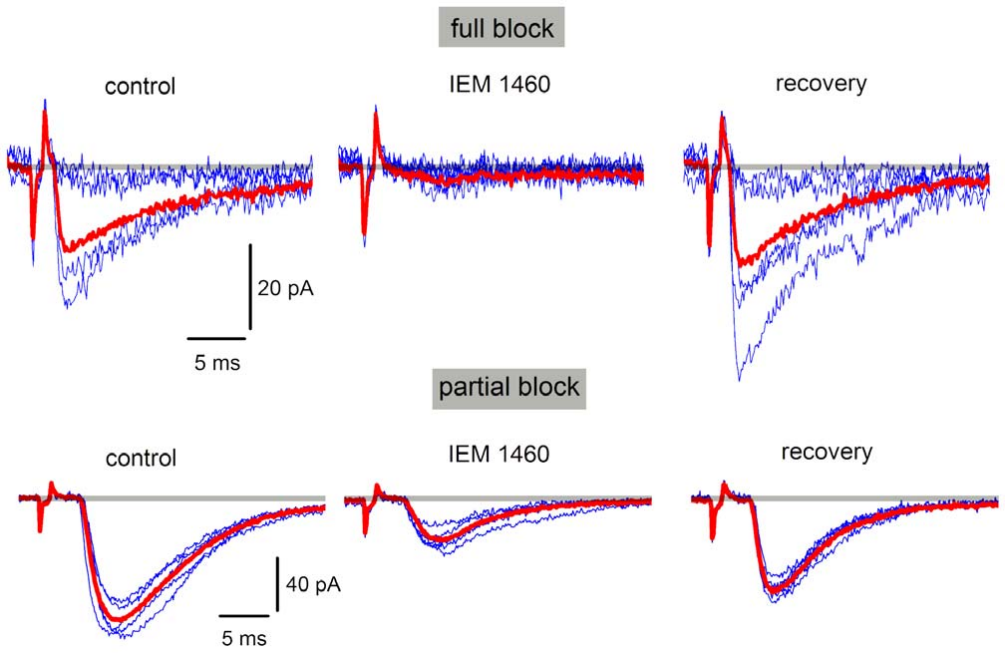
Effect of a CP-AMPAR blocker on evoked EPSCs. EPSCs evoked in two different cells by stimulation of SG EINs. EPSCs were recorded in the absence and presence of the specific CP-AMPAR blocker IEM 1460 (20 µM). The EPSCs were completely blocked in some neuron pairs (top), whereas the blockade was only partial in other cases (bottom). The drug was washed-out (recovery) after the steady-state level of block has been reached. Red traces are averages of five consecutive EPSCs (blue).

The AMPA/kainate receptor blocker CNQX (10–20 µM) was tested in 10 pairs. A complete EPSC block was obtained in 5 cases (full recovery, n = 4). In the remaining pairs, both blocks (by 43.4±11.4%, n = 5) and recoveries were incomplete, probably, due to diffusion problems for some deep synapses in the spinal cord slice. The AMPAR blocker GYKI-52466 (100 µM) was tested in 21 pairs. The EPSC block was complete in 14 cases (recovery, n = 12). In 7 pairs, the block was partial (by 72.3±6.8%) and recovery was obtained in only 2 cases. The specific blocker of CP-AMPARs IEM 1460 (20 µM) was tested in 43 pairs (Fig. 4). In 22 cases, a complete block with recovery was observed. In 15 pairs, the block was partial (by 44.0±7.2%) and the recovery to >80% was obtained in 9 cases. In the remaining 6 connections, the EPSCs were insensitive to IEM 1460. The kainate receptor blocker SYM 2081 (1 µM) was tested in 25 pairs. In no case was a full block observed. A partial block (by 43.2±10.1%) was seen in 6 pairs but recovery was obtained in only 2 cases. In 19 pairs, SYM 2081 had no effect. The NMDAR blocker D-AP5 (50 µM) had no effect on EPSCs recorded in 1 mM Mg^2+^-containing ACSF (n = 5), and we also did not see effect of the blocker when tested in Mg^2+^-free ACSF [4]. The EPSCs were not reduced by a mixture of the GABA_A_ receptor blocker picrotoxin (100 µM) and the glycine receptor blocker strychnine (1 µM) (n = 5).

These tests have shown that transmission from an SG EIN involves both the CP- and CI-AMPARs.

### Time Course of EPSCs

**Figure 5:**
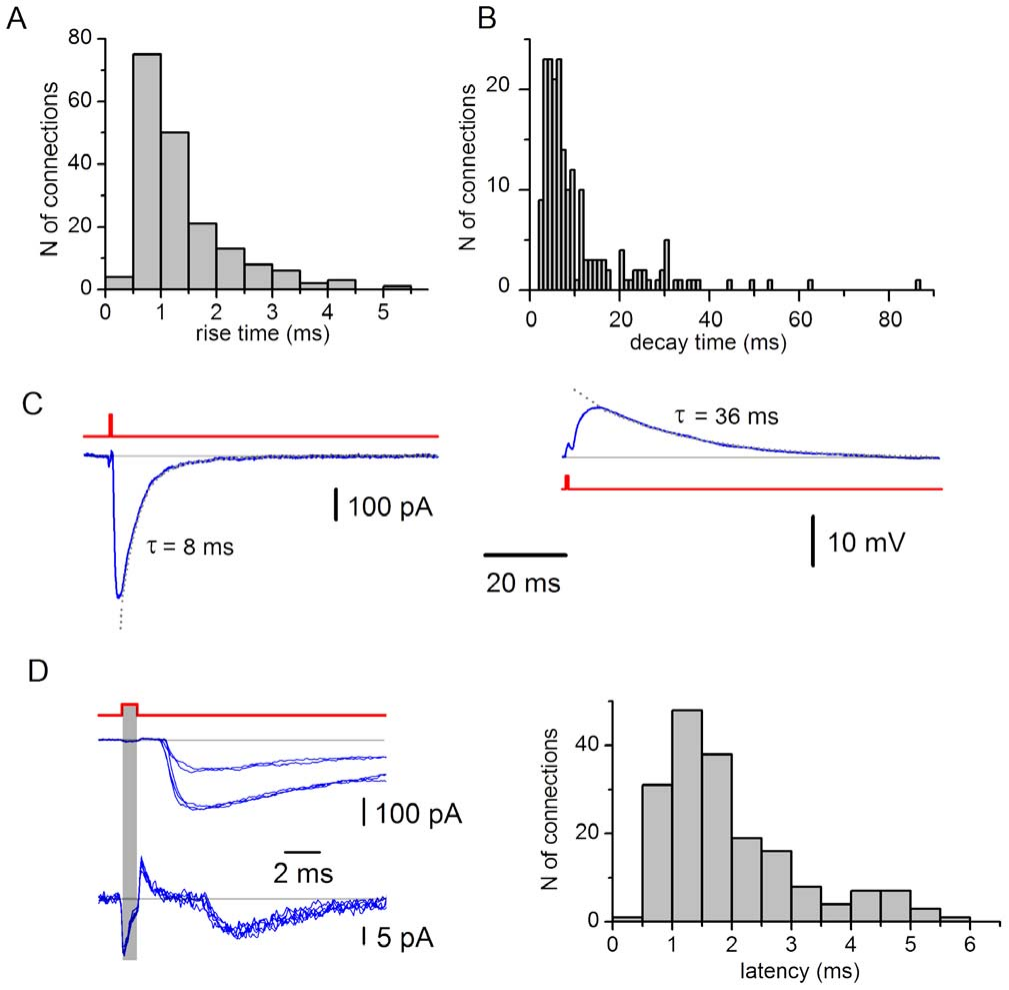
Time course and latency of monosynaptic EPSCs. A, the distribution of the rise times from 10% to 80% of the EPSC amplitude (n = 183). The rise time was measured in averaged (15–30) traces. B, the distribution of the EPSC decay time constants (n = 194; 183 connections). In 11 connections, the decay was fitted with two exponentials. C, monosynaptic EPSC (−80 mV) and EPSP (−70 mV) elicited in the same neuron by stimulating an SG EIN. The decays are fitted with exponentials. D, the distribution of the latency times (n = 183). Latencies were measured as an interval between the end of the stimulation (grey bar) and the beginning of the EPSC measured at 10% of the amplitude. Recordings (each family of traces contains 5 EPSCs evoked by consecutive stimulations) are shown for two connections with different latencies.

We measured the rise time and the decay time constant of EPSCs evoked by stimulating SG EINs. The rise time ranged from 0.46 ms to 5.4 ms giving the histogram peak at 0.5–1.0 ms (Fig. 5A). The EPSC decay was analysed by fitting traces with one (n = 172) or two (n = 11) exponential functions (Fig. 5B). Eighty-four percent of the decay time constants were in the interval between 2 ms and 17 ms. The time courses of EPSCs and EPSPs are illustrated in Fig. 5C. In all connections, EPSCs decayed faster than EPSPs. In 8 pairs analysed, the EPSC and EPSP decay time constants were 8.9±0.7 ms and 35.8±2.1 ms, respectively.

### Variable EPSP Latencies Explained by Different Location of the Synapses along the Dendritic Tree

The latency times ranged for different connections from 0.48 ms to 5.5 ms (Fig. 5D; mean 1.9±0.1 ms, n = 183).

For the reason given in Discussion, the longest latencies observed could not be explained by spike propagation in the presynaptic neuron and transmitter release. Therefore, computer simulations were done to test whether additional delay could be caused by the electrotonic EPSC propagation in the postsynaptic neuron, when release occurred at distal synapses. We also studied whether different dendritic locations of the synapse can explain observed variations of the rise and decay kinetics.

**Figure 6:**
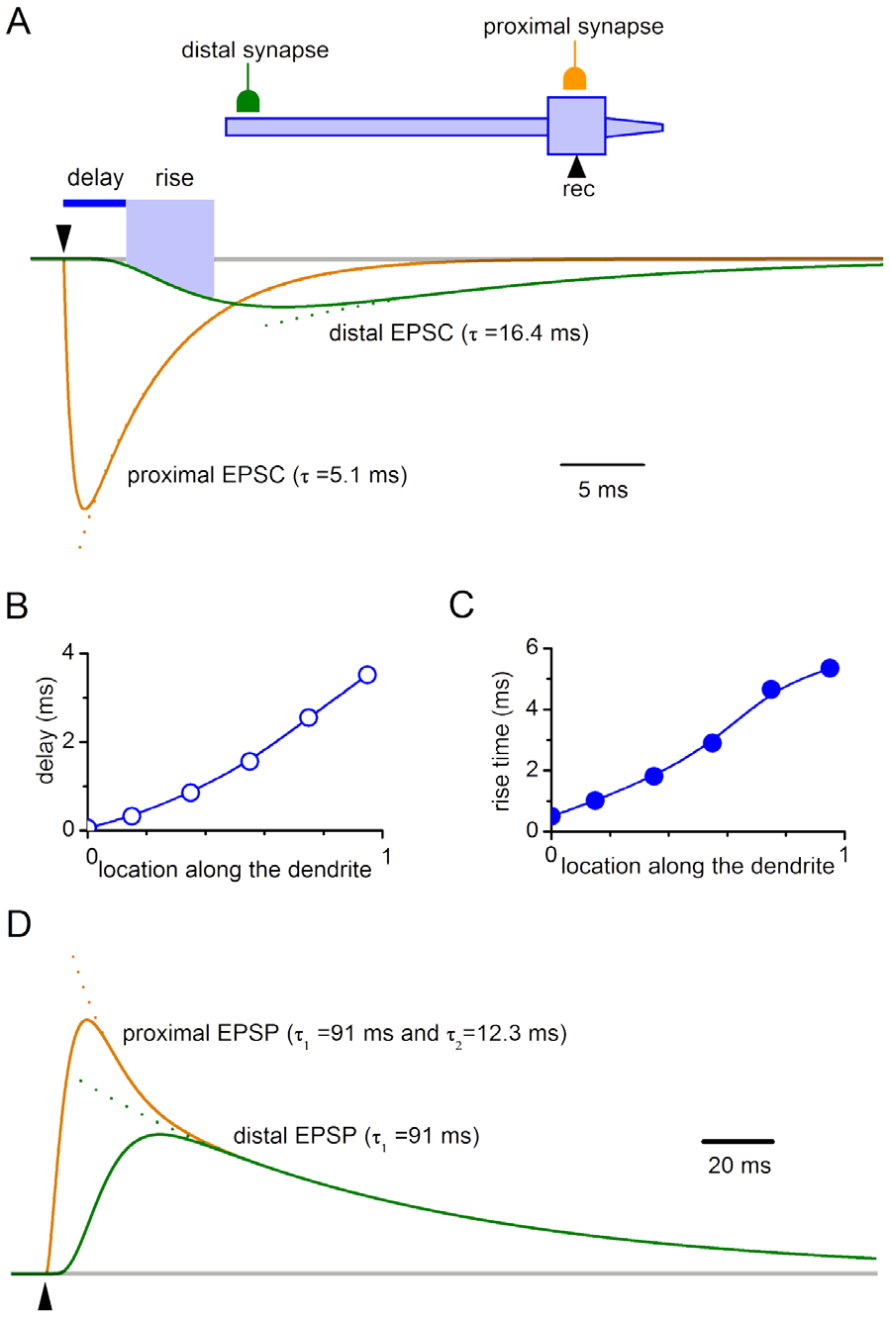
Simulation of proximal and distal inputs. A, simulation of proximal (soma) and distal EPSCs seen with electrode placed at the soma. Distal synapse is at dendrite 0.95; assuming 0 as the proximal end and 1 as the distal end. Both synapses have the same kinetics and g_M_ (9.2*10^−5^ µS). The EPSC decay was fitted with an exponential. B, the electrotonic delay of the EPSC propagation to the soma as a function of the synapse location at the dendrite. The delay was measured to the moment when the EPSC seen at the soma reached 10% of its amplitude. C, the EPSC rise time (from 10% to 80%) as a function of the synapse location. D, simulation of proximal (soma) and distal (dendrite 0.95) EPSPs seen at the soma. The decay of the distal EPSP is fitted with one exponential, whereas that for the proximal EPSP with two exponential functions.

Recording was simulated for the electrode placed at the soma, while a synapse was moved along the somatodendritic domain (Fig. 6A). In VC, the proximal EPSC showed fast decay with the time constant (∼5 ms) resembling τ_D_ of the kinetic scheme describing synapse (see Methods). When this synapse was moved distally (dendrite 0.95), the EPSC became slower (decay time constant, 16.4 ms) and smaller. The electrotonic delay of the EPSC increased from 0.05 ms for the somatic synapse to 3.51 ms for the distal (dendrite 0.95) synapse (Fig. 6B). The EPSC rise time also changed with the synapse location from 0.5 ms (soma) to 5.35 ms (dendrite 0.95) (Fig. 6C). In CC (Fig. 6D), the proximal EPSP had two components in the decay kinetics, of which the slow one corresponded to the membrane time constant of the model neuron. The distal EPSP had slower rising phase and monoexponential decay. The electrotonic delays were 2.2 ms and 8.1 ms for the proximal and distal EPSPs, respectively.

Thus, our experiments have shown that SG EINs elicit fast EPSCs. The variation range of their kinetics, in the majority of cases, can be explained by different dendritic locations of the synapse. Electrotonic propagation in the postsynaptic neuron can substantially increase the latency of distal EPSCs.

### Detection of Distal Dendritic Inputs at the Soma

VC is widely used as a principal mode for recording spontaneous and evoked synaptic activity in the superficial dorsal horn [4], [14], [45]–[48]. However, its applicability for detection of weak distal, i.e. inhibitory, inputs has recently been questioned [49], [50]. Lower signal-to-noise ratio in VC has been assumed as major reason for the different proportions of excitatory and inhibitory connections seen in CC [2], [3], [50] and VC [4] experiments. Therefore, we compared the resolution power of both techniques in detecting distal synapses. Recording of weak distal inputs was assumed to be affected by the membrane noise, R_IN_ and the driving force for the carrier ion. Effects of the first two factors were studied experimentally and the last one is analyzed in Discussion.

**Figure 7:**
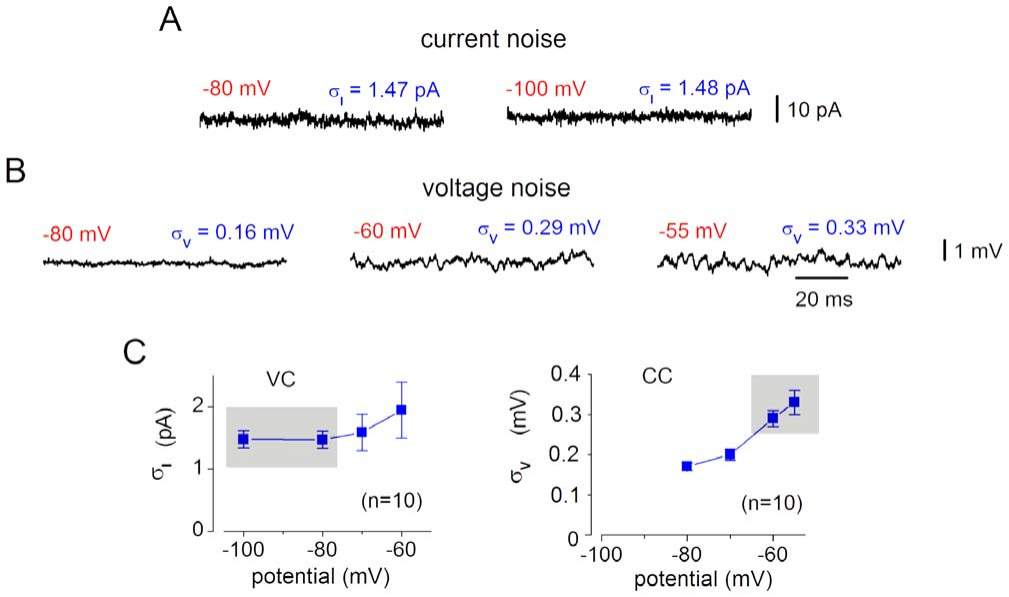
Membrane noise in SG neurons. A, the current noise in VC (filter, 3 kHz). The σ_I_ values represent the means of measurements in 10 SG neurons. B, the voltage noise in CC. The σ_V_ values are the means (n = 10). Note increase in the voltage-noise at -60 mV and −55 mV due to subthreshold activity of Na^+^ channels [36]. C, σ_I_ and σ_V_ as functions of membrane potential. VC and CC data are from the same neurons. Grey boxes indicate the current and voltage noise at potentials used for comparison with other studies.

Membrane noise was measured at potentials used for recording of synaptic inputs. VC identification in this study and [4] was done at −80 mV and −100 mV (a pulse to −60 mV was only applied to see the IPSC reversion). CC identification in [2], [3], [50] was done at −50 mV and −60 mV. Therefore, the current noise at −80 mV and −100 mV was compared with the voltage noise at −60 mV and −55 mV in the same 10 SG neurons. In VC, the current noise at −80 mV (σ_I_ = 1.47±0.14 pA) and −100 mV was low (Fig. 7A) but increased with depolarization (Fig. 7C) due to subthreshold activity of voltage-gated Na^+^ and K^+^ channels [36], [51]. In CC, the voltage noise also increased with depolarization to −60 mV (σ_V_ = 0.29±0.02 mV) and −55 mV (Fig. 7B and C), where it showed a typical component with a frequency of >100 Hz caused by the subthreshold activity of Na^+^ channels [36], [51]. At all potentials analyzed (Fig. 7C), our σ_I_ and σ_V_ values were in good agreement with those from specialized studies [36], [51].

**Figure 8:**
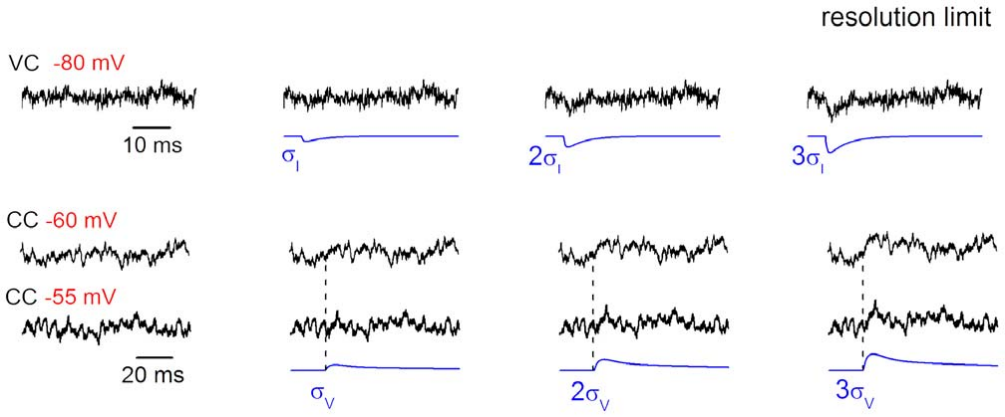
Resolution limits. Determination of the resolution limits for VC and CC recordings. Simulated (noise-free) EPSCs and EPSPs were added to the original recordings of current noise at −80 mV and voltage noise at −60 mV and −55 mV, respectively. The amplitude of the EPSC and EPSP was expressed in the corresponding σ (σ, 2σ and 3σ) calculated for each individual recording. Synaptic responses were considered as resolved at the 3σ level.

To determine the resolution limits, which also depend on spectral compositions of responses and noise, simulated EPSC and EPSP were added to original recordings of current and voltage noise, respectively (Fig. 8). In VC, the EPSC could be already detected at 2σ_I_ amplitude, and was clearly resolved at 3σ_I_ amplitude. Detection was more difficult in CC, where the Na^+^-channel-dependent component of the membrane noise [36] masked the EPSP. Reliable detection of EPSP could not be done for amplitudes below 3σ_V_. Therefore, the resolution limit of 3σ was assumed for both VC and CC. Our data (Fig. 7C) suggest that the smallest EPSC adequately resolved at −80 mV has amplitude of ∼5 pA. This limit could be also validated by inspecting the traces in Fig. 5D (lower traces) and Fig. 15A. Similar signal-to-noise ratio for the EPSP recorded at −60 mV is achieved at 0.9 mV.

**Figure 9:**
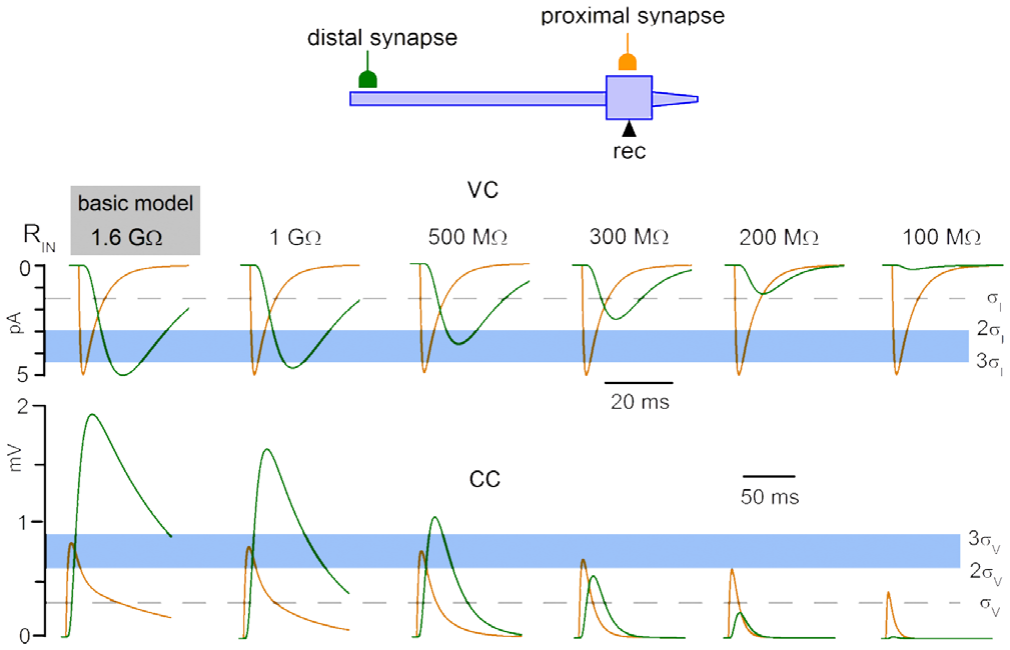
Effect of R_IN_ on the resolution of distal inputs. Computer model of SG neuron was used to simulate somatic recordings of the distal (dendrite 0.95) and proximal (soma) inputs at varying R_IN_. The g_M_ values, 9.2*10^−5^ µS for the proximal synapse and 5*10^−4^ µS for the distal synapse, were adjusted in the basic model to give EPSCs of 5 pA. Horizontal lines indicate the σ, 2σ and 3σ levels of resolution. The σ values were from our experiments: σ_I_ = 1.47 pA (−80 mV) and σ_V_ = 0.29 mV (−60 mV).

R_IN_ affects detection of distal inputs because an increase in the membrane conductance associated with its drop shunts EPSC/EPSP propagation to the soma. The mean R_IN_ is >1.6 GΩ in the present study and [4] but drops to 158±68 MΩ [26] when Ca^2+^-chelator-free pipette solution is used [2], [3], [26]. Therefore, we studied effect of R_IN_ variation from 1.6 GΩ to 100 MΩ in the SG neuron model with the distal and proximal synapses (Fig. 9). Synapses were adjusted in the basic model to give EPSCs of 5 pA (VC, 1.6 GΩ). This model of the smallest distal and proximal inputs resolved in our experiments (>3σ_I_) was used to study effects of the R_IN_ drop in both VC and CC. In VC, R_IN_ had virtually no effect on the proximal EPSC which always remained >3σ_I_ (Fig. 9, VC). However, the distal EPSC became <2σ_I_ at 300 MΩ and <σ_I_ at 200 MΩ. In CC, the proximal EPSP was <3σ_V_ at 1.6 GΩ and became <2σ_V_ at 100 MΩ. The distal EPSP was >3σ_V_ and could be well resolved at R_IN_>500 MΩ. For R_IN_ <500 MΩ, the distal EPSP became <2σ_V_ at 300 MΩ and <σ_V_ at 200 MΩ, and could no longer be considered as resolved. Thus, the smallest distal EPSC recorded under our conditions could not be adequately resolved in VC or CC experiments with R_IN_<500 MΩ. The amplitudes of simulated EPSCs and EPSPs expressed in the corresponding σ (Fig. 9) allowed a comparison of the signal-to-noise ratios for recording distal inputs in high-R_IN_ VC and low-R_IN_ CC experiments. The signal-to-noise ratio in VC at 1.6 GΩ (3.4 σ_I_) was 1.8, 4.5 and 34 times higher than in CC at 300 MΩ (1.9 σ_V_), 200 MΩ (0.76 σ_V_) and 100 MΩ (0.1 σ_V_), respectively.

This analysis has shown that, for both proximal and distal synaptic inputs, high-R_IN_ VC has higher signal-to-noise ratio than low-R_IN_ CC. Furthermore, the efficacy of distal EPSPs is reduced with the drop in R_IN_.

### Efficacy of Synaptic Transmission

Transmission efficacy was studied in 22 connections. The postsynaptic neuron was kept in CC at a potential close to its resting potential of −70 mV, while the presynaptic SG EIN was stimulated each 20 s.

**Figure 10:**
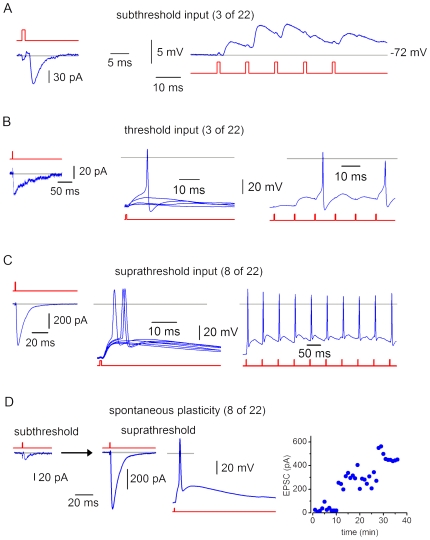
Efficacy of synaptic transmission. A, EPSCs (−80 mV) and EPSPs recorded in a neuron with the subthreshold input from an SG EIN. The EPSP summation was evoked by 5 stimulations of the presynaptic SG EIN at 100 Hz. B, connection with the threshold input. Some single EPSPs elicited spikes. The EPSP summation evoked by 6 stimulations of the presynaptic SG EIN at 100 Hz elicited two spikes. C, connection with the suprathreshold input. Single EPSPs frequently initiated spikes. Repetitive firing could be induced in the postsynaptic neuron by the EPSP summation (the SG EIN was stimulated at 20 Hz). D, recordings from the connection which showed spontaneous plasticity. Each point in the graph represents the EPSC amplitude obtained by averaging 3 consecutive episodes recorded within 1 min. Grey lines, 0 mV or 0 pA.

In 3 connections (Fig. 10A), the input from the SG EIN was classified as subthreshold and neither single nor summed EPSPs elicited spikes. The largest single or summed EPSPs were 5–8 mV. The mean EPSP failure rate was 0.46±0.06 (n = 3; range, 0.37–0.58; 56 episodes analysed). The mean EPSCs ranged from 15 pA to 90 pA.

In 3 connections (Fig. 10B), the input was at the level of firing threshold with the probability of eliciting spike by single EPSPs below 20% (9.7±3.1%, n = 3). Spikes could be also evoked by the EPSP summation. The largest EPSPs which did not evoke spikes were 11–16 mV. The EPSP failure rate was 0.38±0.24 (n = 3; range, 0.13–0.84; 258 episodes analysed). The mean EPSC amplitudes were 37–52 pA.

In 8 connections (Fig. 10C), the input was classified as suprathreshold and single EPSPs initiated spikes with a probability of 67.4±7.5% (n = 8). The EPSP summation could evoke multiple spikes. The largest EPSPs which did not evoke spikes were 8–25 mV. The EPSP failure rate was 0.35±0.10 (n = 8; range, 0–0.91; 649 episodes analysed). The mean EPSC amplitudes were 37–399 pA.

In the remaining 8 connections, spontaneous synaptic plasticity was observed. In the experiment shown in Fig. 10D, the EPSC was small during the first 10 min of recording (mean, 20 pA; first 15 EPSCs) but then dramatically increased (mean, 610 pA; last 15 EPSCs). Following this, single EPSPs became suprathreshold. In these connections, the EPSC amplitude increased by a factor of 8.5±3.3 (n = 8).

Thus, an SG EIN evokes substantial depolarization of the postsynaptic neuron and individual or summed EPSPs can frequently elicit spikes. Moreover, synapses of SG EINs showed plasticity, which was studied in the following experiments.

### Short-Term Plasticity

**Figure 11:**
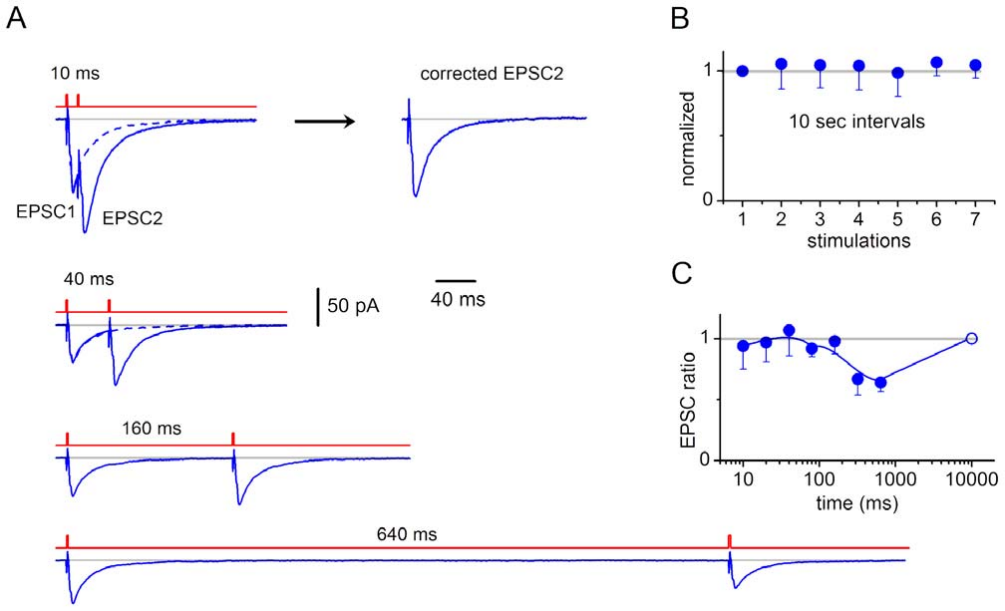
Short-term plasticity. A, EPSCs (−80 mV, averages of 24 traces) elicited by paired-pulse stimulations of a presynaptic SG EIN. Intervals between the paired stimulations were 10 s. The intervals between the pulses in the pair varied from 10 ms to 640 ms. At short intervals, the second EPSC in the pair was corrected for the overlap with the first EPSC as shown for 10 ms. For this, the first EPSC from the trace without overlap (at 640 ms) was scaled to fit the peak of the first EPSC with overlap and then was subtracted from it. B, test for stability of EPSCs at stimulations with 10 s intervals. For each connection, the amplitudes of the first EPSCs (non-averaged) in the first 7 paired stimulations were plotted (the first EPSC was normalized to 1). Each point shows the mean value for 7 connections. C, the ratio between the peak amplitudes of the second and first EPSCs as a function of the interval. For each connection (n = 7), the ratio was calculated from the average of 10–36 traces. The point at 10 s was set to 1 according to data from B.

Short-term plasticity (sub-second range) was induced in 7 connections using a standard paired-pulse protocol (Fig. 11A). No interaction between the paired responses was seen for intervals up to 160 ms (Fig. 11C). At longer intervals (320 ms and 640 ms), a depression of the second response was observed (0.64±0.08, n = 7; at 640 ms, P<0.001). The failure rate for the first EPSC was 0.37±0.14 (n = 7) and for the second EPSC at 320 and 640 ms intervals (pooled) was 0.44±0.13 (n = 14). Thus, the change in the failure rate could not explain the depression observed for these intervals.

### Long-Term Plasticity

**Figure 12:**
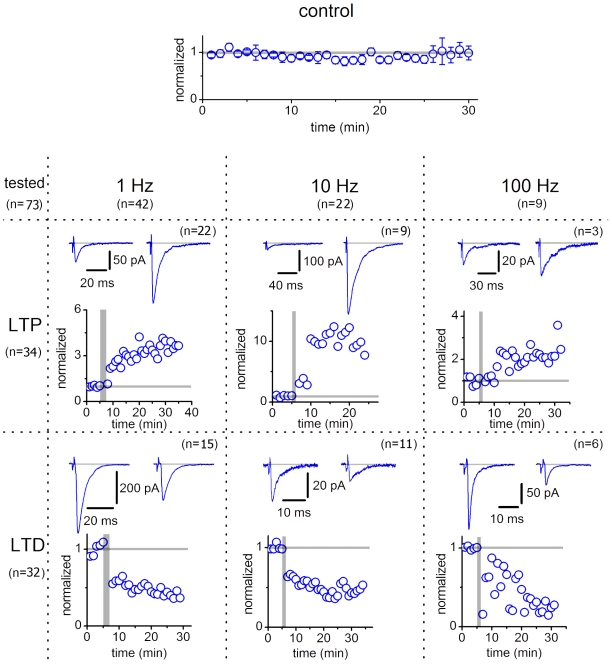
Long-term plasticity in synapses of SG EINs. Control, the postsynaptic neuron was in VC at −80 mV and the presynaptic SG EIN was stimulated each 20 s. EPSCs obtained by 3 consecutive stimulations were averaged and presented as one point. The control graph shows the average of normalized values (n = 6). LTP and LTD were induced by three different protocols of stimulations at 1 Hz, 10 Hz and 100 Hz. During application of the induction protocol, the postsynaptic neuron was switched to CC. Illustrations of induced plasticity are based on recordings from individual neurons. Before induction of plasticity (indicated by the vertical grey bar), each connection was stimulated during 5 min. The mean EPSC amplitude measured before induction was normalized to 1. The times, at which the changes in the EPSC amplitudes are reported in the text, were calculated from the end of the application of the induction protocol.

Different forms of long-term plasticity (tens of minutes) was induced by stimulating SG EINs at 1 Hz, 10 Hz and 100 Hz. Before and after the induction protocol application, the EPSCs were evoked each 20 s. At this interval, EPSCs remained stable in 6 of 8 control experiments (Fig. 12), while in 2 cases spontaneous potentiation occurred as reported above (not shown). Induction protocols were applied in 73 connections and the plasticity was considered as induced (n = 66) if its appearance correlated with the induction protocol application and the EPSC amplitude changed by more than 25%.

Stimulation at 1 Hz applied for 2 min was tested in 42 pairs. In 22 cases, it caused a significant and irreversible increase in synaptic strength observed for 12–52 min until the end of the recording (Fig. 12, LTP, 1 Hz). The EPSC increased by a factor of 2.9±0.4 (n = 22, P<0.001, range from 1.27 to 6.6) at 12 min and 3.2±0.4 (n = 14, P<0.001) at 30 min. In 15 pairs, the protocol induced depression (Fig. 12, LTD, 1 Hz) recorded for 14–50 min. The EPSC amplitude reduced to 0.41±0.05 (n = 15, P<0.001, range from 0.11 to 0.64) at 14 min and 0.36±0.05 (n = 8, P<0.001) at 30 min. In the remaining 5 pairs neither LTP nor LTD were induced.

Stimulation at 10 Hz applied for 1 min was tested in 22 pairs, in 9 of which potentiation was induced (Fig. 12; LTP; 10 Hz) and lasted for 12–50 min. The EPSC was increased by a factor of 4.7±1.1 (n = 9, P<0.001, range from 1.41 to 10.1) at 12 min and 4.0±1.9 (n = 5, P<0.2) at 30 min. In the remaining 11 pairs, depression was induced and lasted for 10–40 min (Fig. 12; LTD; 10 Hz). The EPSCs decreased to 0.51±0.04 (n = 11, P<0.001, range from 0.33 to 0.7) at 10 min and 0.43±0.08 (n = 7, P<0.001) at 30 min.

High-frequency stimulation (100 Hz applied for 1 s, repeated 3 times with 1 s intervals) was tested in 9 pairs. In 3 cases, LTP was induced and observed for 11–46 min. The EPSC increased by a factor of 2.2±0.3 (n = 3, P<0.05, range 1.82 to 2.68) at 11 min and 2.3±0.5 (n = 2, P<0.2) at 30 min. In 6 pairs, we observed LTD (15–35 min). The EPSCs decreased to 0.36±0.05 (n = 6, P<0.001, range from 0.2 to 0.48) at 15 min and 0.21±0.02 (n = 3; P<0.001) at 30 min.

Thus, synapses of SG EINs show diverse forms of functional plasticity.

### Monosynaptic EPSCs Are Evoked by Transmitter Release in Multiple Synapses of the Same Axon

**Figure 13:**
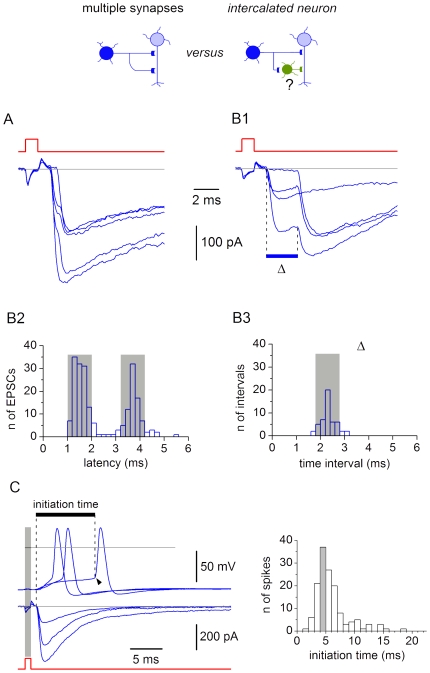
Composite EPSCs evoked by transmitter release in multiple synapses. A, EPSCs with a fast transition on the rising phase. The interval between the components (Δ) was 0.4 ms. B1, EPSCs with Δ of 2.3 ms. B2, latency distributions for individual components of the EPSC from B1. Width of grey boxes is 1 ms. The histogram includes the episodes with both components and one of the components (n = 212). B3, distribution of Δ for the composite EPSC from B1. The histogram is based on 50 episodes with both components. The grey box has a width of 1 ms. C, spike initiation by EPSPs. The moment of the transmitter release (left vertical dashed line) was determined in VC, where the measurement was more precise. In CC, the postsynaptic neuron was at −70 mV and spikes were evoked by stimulating the presynaptic SG EIN. Beginning of the spike depolarization (arrowhead) was considered as the moment of the spike initiation in the AIS (see Fig. 3). Right, distribution of the spike initiation times (n = 142), calculated as an interval between the transmitter release and the spike initiation moments. Bin width, 1 ms. The largest bin (grey) contains 37 of 142 events.

In the majority of pairs (152 of 183), the EPSCs were composite and at least two components could be distinguished on their rising phase. In some cases, transitions between the components were seen as inflexion points (Fig. 13A), however, in other cases, the components were clearly separated in time (Fig. 13B1). In all these pairs, we also recorded individual components (Fig. 13A and B1) which had the same latencies as in the composite EPSC. The time interval between the first two components of the composite EPSC (Δ) remained constant during recording and ranged from 0.3 ms (3 kHz filter) to 3.1 ms.

Since a composite EPSC was elicited by one spike, one could rule out the possibility that the components appeared due to variation in the presynaptic spike initiation time. Two hypotheses could explain the appearance of composite EPSCs. 1) The axon of an SG EIN forms multiple synapses on a postsynaptic neuron; in this case, simultaneous activation of synapses with different electrotonic locations elicits EPSCs reaching the soma with different delays. 2) In addition to a monosynaptic connection, the neurons are also connected via an intercalated neuron; different conduction times in these pathways might also explain appearance of composite EPSCs. Although an involvement of an intercalated neuron was unlikely in connections with Δ<1 ms (Fig. 13A), it could not be excluded for those with larger Δ (Fig. 13B1). Therefore, we analysed 5 connections with large Δ to distinguish between the hypotheses.

For the composite EPSC from Fig. 13B1, the latency distributions for both components was constructed and showed two distinct peaks (Fig. 13B2). The width of each peak, corresponding to the latency variation for each component, was within 1 ms (grey boxes). Thus, each EPSC component could be independently identified as monosynaptic based on the standard criterion of latency stability. We also analyzed the Δ variation in the composite EPSCs (Fig. 13B3). With the exception of 6 measurements, all values were within the 1 ms range (grey box). Similar variations (<1 ms) of Δ was seen in all 5 analysed connections with large Δ (2.5±0.3 ms, n = 5). The following analysis was done to test whether such small Δ variations can be consistent with the idea of involvement of the intercalated neuron.

The delay on the intercalated neuron consists of the time taken for the presynaptic spike to begin the EPSP (synaptic delay), the time necessary for the EPSP to grow and initiate the spike (initiation time), and the time needed for the spike propagation along the axon of the intercalated neuron (propagation time) [52]. Since the synaptic delay and the propagation time are short (<1 ms), the initiation time was assumed to be the major contributor to the conduction delay. In addition, the initiation time should vary with the spontaneous changes in the quantal transmitter release from the presynaptic neuron. Therefore we analysed the spike initiation time and its variation to rule out the possibility of involvement of the intercalated neuron.

For these experiments, 9 connections were chosen with suprathreshold EPSPs and rapid spike initiation. The postsynaptic neurons had high R_IN_ (2.2±0.4 GΩ, n = 9). In the connection shown in Fig. 13C, the EPSP failure rate was low (0.10) and the probability of spike initiation by a single EPSP was high (62%). In VC, the EPSC amplitude varied from episode to episode indicating variation in the transmitter release (Fig. 13C). In CC, the fluctuations in the quantal transmitter release caused variation of the spike initiation time. The histogram showed a broad distribution of the initiation times from 1.9 ms to 18.2 ms (mean 6.2±0.3 ms, n = 142). This histogram was binned at 1 ms which is usually considered as the largest latency variation allowed for the monosynaptic connection. The maximum bin (grey) contained only 26% of events, indicating that it was not possible to select a 1 ms range of delay variation containing more than about one-fourth of initiated spikes. The mean initiation time in all 9 connections was 8.9±2.1 ms. The largest bin contained 28±3% (n = 9) of events, indicating that the vast majority of synaptically evoked spikes showed the initiation time variation >1 ms.

These experiments have shown that the spike initiation time and its variation in the intercalated neuron should be substantially larger than Δ and its variation in the composite EPSC. Therefore, an existence of multiple synapses between the neurons is likely explanation for the composite EPSC.

**Figure 14:**
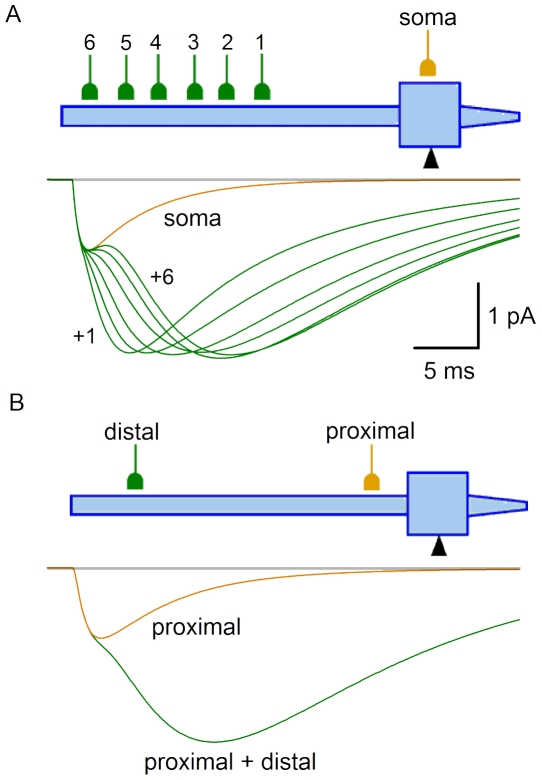
Modelling of composite EPSC. A, the proximal synapse was at the soma (g_M_ = 2*10^−5^ µS), while the distal synapse was moved along the dendrite and its g_M_ was changed to keep the amplitude of composite EPSC. Points 1–6 and the g_M_ values were: dendrite 0.41 and 1.3*10^−4^ µS, dendrite 0.51 and 1.7*10^−4^ µS, dendrite 0.61 and 2.1*10^−4^ µS, dendrite 0.71 and 2.3*10^−4^ µS, dendrite 0.81 and 2.5*10^−4^ µS, and dendrite 0.91 and 2.5*10^−4^ µS, respectively. A simple EPSC was simulated by activating the somatic synapse only (trace, soma). Simultaneous activation of somatic and dendritic synapses gave composite EPSCs (traces, soma+1 to +6). B, simulation with two dendritic synapses, proximal (dendrite 0.11, g_M_ = 3*10^−5^ µS) and distal (dendrite 0.81, g_M_ = 2.3*10^−4^ µS). Simultaneous activation of these synapses gave a composite EPSC seen at the soma.

We also tested whether composite EPSCs could be modelled by simultaneously activating synapses with different somatodendritic locations. In the simulation shown in Fig. 14A, one synapse was positioned at the soma, while the other one was moved along the dendrite (points 1–6). Simultaneous activation of both synapses gave composite EPSCs, in which Δ increased with the distance between the synapses. A composite EPSC could also be simulated for two dendritic synapses (Fig. 14B). These simulations confirmed that the composite EPSCs could be evoked by the activation of multiple synapses of one presynaptic neuron distributed along the somatodendritic domain of the postsynaptic neuron. In this case, Δ reflects the electrotonic distance between the synapses.

**Figure 15:**
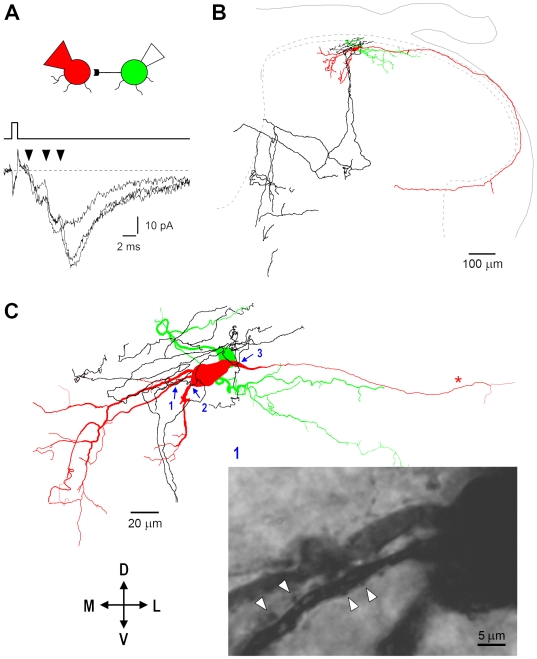
Reconstruction of synaptically connected neurons. A, composite EPSCs (−80 mV) with at least three components (arrowheads). The postsynaptic neuron was filled with biocytin in whole-cell mode, while the presynaptic one through the cell-attached pipette [28]. B, both cell bodies were in the SG. The axon of the postsynaptic neuron (red) ran along the dorsal surface of the grey matter, gave a collateral in the lateral column, and turned medially to re-enter the grey matter. The presynaptic cell axon (black) formed a dense network in the vicinity of the cell bodies and divided into two major branches which travelled to deeper laminae and turned towards the dorsal grey commissure. Soma and dendrites of the presynaptic neuron are shown in green. C, a number of close appositions between the varicosities of the presynaptic axon and the dendrites and soma of the postsynaptic neuron were detected in regions 1–3 (blue arrows). Some of them are shown (arrowheads) on the photomicrograph of the region 1. Objective; 100x (oil-immersion), numerical aperture 1.25.

Finally, in two connections with composite EPSCs we have succeeded to obtain a detailed filling with biocytin of both the axon terminals of the presynaptic SG EIN and the dendritic arbores of the postsynaptic neuron. In the connection shown in Fig. 15, the composite EPSC had three components (Fig. 15A). The presynaptic cell axon formed a dense network in the vicinity of the postsynaptic cell body (Fig. 15B). At higher magnification (Fig. 15C), a number of close appositions (indicating putative synaptic contacts) between the varicosities of the presynaptic axon and the dendrites and soma of the postsynaptic neuron were detected in regions 1–3. In the other labelled connection, three putative synaptic contacts have also been revealed. Although it was not possible to estimate the electrotonic distance between the synapses and their total number, or to attribute the groups of synapses to the components of a composite EPSC, the labelling experiments suggested that multiple synaptic contacts between an SG EIN and a postsynaptic neuron can form the anatomical basis for the composite EPSCs.

Thus, paired recordings, computer simulation and biocytin labelling indicated that the majority of the monosynaptic EPSCs are likely to be generated by transmitter release in multiple synapses formed by the axon of an SG EIN on a postsynaptic neuron.

### Probability of Release in an Individual Synapse

In the experiments described above, we calculated the failure rates for composite EPSC/EPSP to characterize transmitter release in multiple synapses. In some connections, however, it was possible to analyse individual components of the composite EPSC and estimate the release probability of the corresponding synapses. We analysed 10 such synapses in 5 connections. The release probability in these synapses was 0.51±0.07 (n = 10). If two such synapses were single and independent, the expected failure rate for their composite response (no synapse activated) would be (1–0.51)^2^ = 0.24. Indeed, the mean failure rate for the bi-component response in these connections was 0.21±0.07 (n = 5). These values suggested that the two synapses were individual and operated independently.

### Role of Individual Synapses in Plasticity

**Figure 16:**
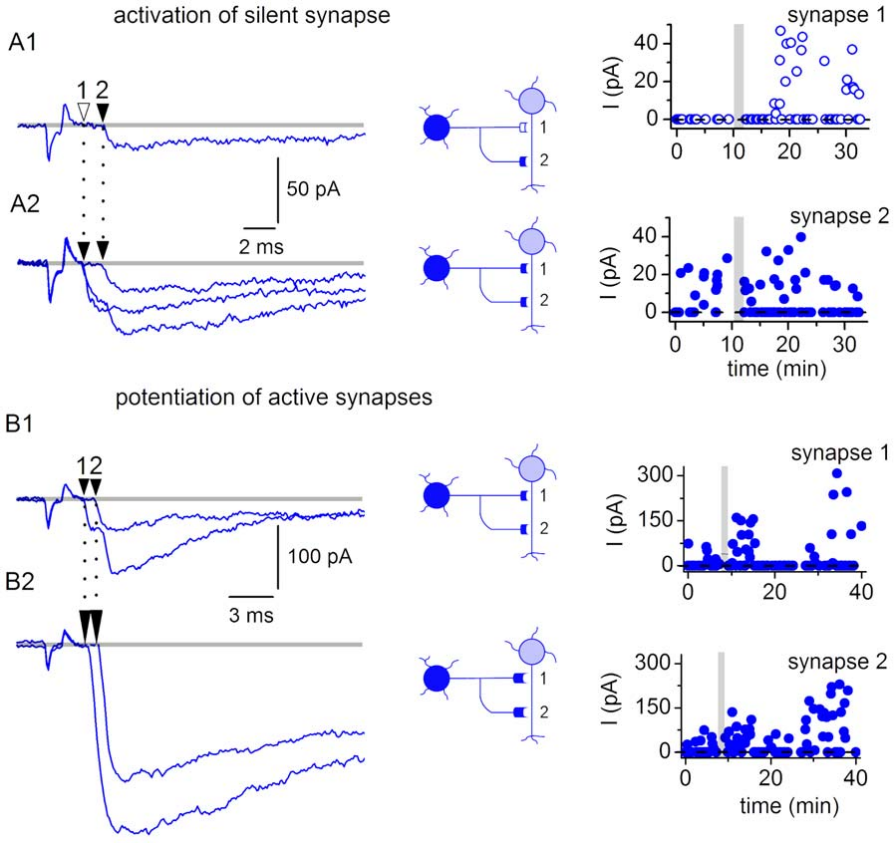
Role of individual synapses in plasticity. A1-2, LTP associated with an activation of a previously silent synapse. A1, EPSC elicited before the application of the induction protocol corresponded to activation of more distal synapse 2. A2, EPSC recorded after induction of LTP (1 Hz protocol). A new component corresponded to activation of more proximal synapse 1. Two plots show the amplitudes of the EPSC components corresponding to synapse 1 and synapse 2 during the experiment. The amplitudes were measured from non-averaged traces. B1-2, LTP associated with an increase in the amplitude of components forming the composite EPSC. Recordings before (B1) and after (B2) the LTP induction (1 Hz protocol). Two plots show the EPSC components corresponding to synapse 1 and synapse 2.

In 10 of 36 connections with induced LTP (Fig. 12) and in 3 of 8 connections with spontaneous LTP (Fig. 10D), some components of composite EPSCs could be well identified and it was possible to follow the changes in properties of individual synapses. In 6 cases, LTP (induced, n = 3; spontaneous, n = 3) was probably associated with an activation of previously silent synapses. One of these connections first showed EPSCs with only one component corresponding to activation of more distal synapse 2 (Fig. 16A1). After the application of induction protocol, a new component corresponding to activation of more proximal synapse 1 appeared (Fig. 16A2). These components could be seen both individually and overlapping (as a composite EPSC). We plotted the amplitudes of the components corresponding to synapse 1 and synapse 2 (Fig. 16A, right). In the episodes with overlapping responses, the EPSC amplitude for synapse 1 was measured from the baseline to the inflection point, and for synapse 2 from the inflexion point to the peak. As one can judge from the plot, the LTP was caused by the activation of previously silent proximal synapse 1, while the distal synapse 2 did not show substantial alteration of activity.

In the remaining 7 connections, the induced LTP was associated with an increase in the amplitude of components forming the composite EPSC. In the connection shown in Fig. 16B1 and B2, the EPSC components corresponding to activation of both the proximal synapse 1 and the distal synapse 2 were potentiated (Fig. 16B, right).

Thus, transmission efficacy can be increased through activation of silent synapses and potentiation of already active synapses.

## Discussion

In this study we have described several novel properties of the neuronal network in the spinal SG. In particular, we have characterized excitatory synapses formed by glutamatergic SG interneurons on postsynaptic neurons located in laminae I-III. The major findings of this study are: 1) short (1 ms) depolarization of ∼10 mV can initiate spike in the AIS of an SG neuron; 2) both CP- and CI-AMPARs are involved in transmission from an SG EIN; 3) the axon of an SG EIN forms multiple synapses on a postsynaptic neuron; 4) synapses of SG EINs show broad somatodendritic distribution in the postsynaptic neuron; 5) high-R_IN_ recording from the postsynaptic neuron is necessary for correct estimation of transmission efficacy; 6) in many cases, release from one SG EIN is sufficient to elicit spike in a postsynaptic neuron; 7) synapses of the SG EINs undergo diverse forms of plasticity.

### Rapid Spike Initiation in SG Neurons

Implementation of independent electrodes for stimulation of and recording from one neuron allowed study of the spike initiation process. Spikes in SG neurons consisted of two components similar to those described in motoneurons [37]–[39]. The fast one was an electrotonic projection of the spike generated in the AIS while the slow one corresponded to the antidromic spike invasion to the axon hillock and soma [42], [43]. Short (1 ms) depolarization of ∼10 mV induced by the cell-attached stimulation was sufficient to trigger spike in the AIS, where the ratio of the Na^+^ conductance to the passive capacitive load is high [43]. Similar thresholds were also obtained for synaptically evoked spikes in paired recordings. Substantially higher firing thresholds, however, were seen in our experiments with intracellular injections of 500 ms pulses (19–34 mV, measured from −70 mV [35]). It is likely that the slow depolarization evoked by a long pulse leads to a partial inactivation of Na^+^ channels and activation of K_A_ channels prior to spike initiation, and therefore, increases the firing threshold and delay.

### Multiple Synapses Formed by the Axon of an SG EIN

Analysis of composite EPSCs, computer simulations and labelling experiments indicated that the axon of an SG EIN forms multiple synapses on a postsynaptic neuron. In 83% of connections, the composite EPSCs showed visible transitions between the components. The true number of EINs forming multiple synapses may be even higher. The filter frequency (3 kHz) used in our experiments did not allow to see transitions faster than 0.3 ms, and responses from synapses separated by a short electrotonic distance could not be distinguished. Furthermore, there are silent synapses between the neurons which may become active under certain conditions. Therefore, formation of multiple synapses on a postsynaptic neuron appears to be a general property of an SG EIN.

The time intervals between the components of composite EPSCs could be explained by the electrotonic distances between the synapses in a postsynaptic neuron. In some cases, however, components with longer latency did not have apparently slower rise predicted from our simulations (see Figs. 13B1 and 15A). Therefore, it is possible that some presynaptic factors, like different axonal conduction time for each synaptic terminal, could also contribute to different latencies of the EPSC components. For example, different spike propagation delays at the branching points of the presynaptic axon could contribute to the intervals between the EPSC components.

Both CP- and CI-AMPARs are involved in transmission from SG EINs. AMPARs are known as main transducers of fast central glutamatergic synapses [53]. In agreement with this, EPSCs evoked by the SG EIN stimulations were fast. The distributions of the rise times and the decay time constants were in the range predicted by the model for the fast synapses located at different electrotonic distances from the soma. The longest EPSC latencies obtained (∼5 ms) could not be explained by the time needed for the presynaptic spike propagation and transmitter release. Assuming the conduction velocity in an unmyelinated axon of 1 m/s and the length of the axon branch connecting neurons of <500 µm (our data), the propagation time could be estimated as <0.5 ms. Allowing 0.5 ms for transmitter release, the conduction delay in the presynaptic axon could be estimated as 1 ms. This time might further increase due to the spike propagation delays at the axon branching points. However, the long latencies observed in our study were likely to be caused by the EPSC propagation in the dendrites of postsynaptic neuron. As modelling predicts, the electrotonic delay varies with the synapse location and it takes >3 ms for the distal EPSC to reach the soma. Thus, the variations in the EPSC kinetics and latencies reflected a broad somatodendritic distribution of synapses in the postsynaptic neuron.

### Transmission Efficacy and Synaptic Plasticity

It is believed that excitatory connections between dorsal horn neurons are weak, and EPSPs of 0.9–2.6 mV [2] and 0.5–8.5 mV [50] are not sufficient to elicit postsynaptic spikes [50]. However, these conclusions were based on low-R_IN_ recording from a postsynaptic neuron, which reduced the amplitude of depolarization induced by activation of a given synaptic conductance (see Fig. 9, CC). Furthermore, dialysis of the presynaptic neuron could affect its capacity of transmitter release in multiple synapses. Small EPSCs (mean, 19 pA) in [50] corresponded to release in a single synapse. Thus, the true efficacy of excitatory transmission in those studies appeared to be underestimated.

Our data show that transmission between dorsal horn neurons can be effective. First, a non-dialysed SG EIN releases transmitter in multiple synapses, thus, increasing effective conductance depolarizing the postsynaptic membrane. Second, a high R_IN_ preserved in postsynaptic neuron allows better conversion of the synaptic conductance into membrane depolarization. Therefore, single EPSPs of >10 mV were regularly recorded. In many cases, input from an SG EIN was sufficiently strong to elicit spike in a postsynaptic neuron kept in CC at −70 mV. In some pairs, an SG EIN could even evoke repetitive firing in the postsynaptic neuron.

R_IN_ in dorsal horn neurons was shown to change with temperature [54]. Assuming that both the leak K^+^ conductance (determining R_IN_) and the glutamate-activated conductance (generating EPSP) have similar Q10 (aqueous diffusion of ions [55]) one can conclude that the ratio between them, and therefore, synaptic efficacy should not change with temperature.

We have found that synapses of SG EINs undergo diverse forms of functional plasticity. LTP could be induced through activation of silent synapses and through potentiation of already active synapses. One cannot exclude, however, that potentiation of the components of a composite EPSC was also caused by recruitment of silent synapses with similar electrotonic location, so that their activation did not result in generation of an apparently new component. It is possible that the activation of silent synapses and the strengthening of already active synapses can cooperate in induction of LTP. This plasticity might be induced by Ca^2+^ influx through CP-AMPARs [56] which are expressed in the superficial dorsal horn [10]–[13] and, as we show, are activated by release from SG EINs. It is also possible that heterogeneity of synaptic properties observed in the present study, like CP- and/or CI-AMPAR expression and plasticity, might correlate with the postsynaptic cell type [56].

### Functional Balance between the Excitatory and Inhibitory Inputs

Reports about the balance between the excitatory and inhibitory connections in the spinal dorsal horn are contradictory. Studies using *in situ* hybridization [8], immunocytochemistry [9] and the high-R_IN_ paired recordings in VC ([4] and this study) showed that the majority of SG neurons are excitatory, whereas inhibitory neurons form a minority. In contrast, the low-R_IN_ CC recordings revealed more numerous inhibitory connections, and the authors suggested that the difference in the results was caused by a better detection of weak distal inhibitory inputs in CC [49], [50]. However, our measurements of the membrane noise have shown that the high-R_IN_ VC has higher signal-to-noise ratio. The last factor which has not been considered so far is the driving force for the inhibitory inputs.

IPSPs in [3] were recorded at a 20 mV driving force (recording, −50 mV; E_Rev_ = −70 mV). IPSCs in [4] and here were recorded at similar driving force for both inward (−100 mV) and outward (−60 mV) currents (E_Rev_ = −82 mV). Thus, analyses of all factors showed that our VC recording has higher detection power. The true reason for lower frequency of excitatory connections in [2], [3], [49] can be a shunt of distal inputs by low-R_IN_ recording. Indeed, the longest latencies of IPSPs (3.3 ms) in [3] and EPSPs (2.1 ms) in [2] were short for CC recording, and indicated that none of inputs reported was distal. A recent study using the laser scanning photostimulation technique revealed that the inhibitory receptors are confined to the narrow peri-somatic region of an SG neuron while the glutamate receptors are more widespread [5]. Therefore, shunt of weak distal inputs [2], [3], [49], which are mostly excitatory [5], led to an underestimation of the number of excitatory connections.

The driving force for inhibitory inputs in [50] is unclear. IPSPs were recorded at −60 mV as hyperpolarizations, although at the Cl^−^ gradient used (20 mM/135.5 mM, E_Cl_ = −49 mV) they had to be depolarizing. This implied a voltage error of >15 mV. Furthermore, identification of monosynaptic IPSPs demands analysis of latency variations for individual traces. The voltage noise at −60 mV [36], [51] does not allow reliable detection of non-averaged IPSPs of <0.5 mV. Therefore, many IPSPs in [50] (smallest 0.1 mV) were below the detection limit. Although in some traces apparent noise was lower than expected at −60 mV, this could mean that the true potential was more negative, for example, due to uncompensated liquid junction potential of 13 mV (calculated according to [32]). This, however, would increase the voltage error to ∼30 mV. Thus, uncontrolled voltages and consideration of IPSPs with amplitudes below the resolution limit allowing tests for monosynaptic connectivity question conclusions about the high percentage of inhibitory connections [50].

In conclusion, we have shown that the EIN is an important processing element in the spinal SG. Its axon forms multiple synapses on the soma and dendrites of a postsynaptic neuron. Many of those synapses have functional CP-AMPARs. The excitatory transmission is frequently effective and an SG EIN can elicit spike in a postsynaptic neuron. The excitatory synapses are dynamic and can change their functional state through diverse forms of plasticity. These properties of the SG EINs are important for understanding the mechanisms of the spinal nociceptive processing.
